# A comparative proteomic study of plasma in Colombian childhood acute lymphoblastic leukemia

**DOI:** 10.1371/journal.pone.0221509

**Published:** 2019-08-22

**Authors:** Sandra Isabel Calderon-Rodríguez, María Carolina Sanabria-Salas, Adriana Umaña-Perez

**Affiliations:** 1 Universidad Nacional de Colombia–Sede Bogotá, Facultad de Ciencias, Departamento de Química, Grupo de investigación en Hormonas, Bogotá, D.C., Colombia; 2 Subdirección de Investigaciones, Instituto Nacional de Cancerología de Colombia, Bogotá, D.C., Colombia; Pacific Northwest National Laboratory, UNITED STATES

## Abstract

Acute lymphoblastic leukemia (ALL) is the most common childhood cancer. Owing to the incorporation of risk-adapted therapy and the arrival of new directed agents, the cure rate and survival of patients with ALL have improved dramatically, get near to 90%. In Latin American countries, the mortality rates of ALL are high, for example in Colombia, during the last decade, ALL has been the most prevalent cancer among children between 0–14 years of age. In the face of this public health problem and coupled with the fact that the knowledge of the proteome of the child population is little, our investigation proposes the study of the plasma proteome of Colombian children diagnosed with B-cell ALL (B-ALL) to determine potential disease markers that could reflect processes altered by the presence of the disease or in response to it. A proteomic study by LC-MS/MS and quantification by label-free methods were performed in search of proteins differentially expressed between healthy children and those diagnosed with B-ALL. We quantified a total of 472 proteins in depleted blood plasma, and 25 of these proteins were differentially expressed (fold change >2, Bonferroni-adjusted P-values <0.05). Plasma Aggrecan core protein, alpha-2-HS-glycoprotein, coagulation factor XIII A chain and gelsolin protein were examined by ELISA assay and compared to shotgun proteomics results. Our data provide new information on the plasma proteome of Colombian children. Additionally, these proteins may also have certain potential as illness markers or as therapeutic targets in subsequent investigations.

## Introduction

Pediatric acute leukemia is a genetic and phenotypically heterogeneous disease. Accordingly to the phenotype, acute lymphoblastic leukemia (ALL) may involve B-cell (B-ALL) or T-cell progenitors (T-ALL) [1]. ALL is the most common childhood malignancy, accounting for 25% of all childhood cancers [2]. Of these cases, 80 to 85% are classified as B-lineage ALL [3,4]. The incidence rate of new cases of childhood ALL increases every year; fortunately, strides have been made in the management of childhood ALL over the past 50 years; this management has resulted in the improvement of cure rates from 10% to 90% in developed countries [2]. However, of the approximately 160,000 children and teenagers diagnosed with cancer every year worldwide, 80% live in low- and middle-income countries (LMICs) where access to quality care is limited and the chances of being cured are low [5]. In addition to the difficulties in diagnosing and treating the disease, as well as inadequate medical facilities and other socioeconomic factors affecting the incidence and survival of patients with ALL in poor countries. The incidence rates of ALL in several Latin American countries are the highest in the world [6]. In Colombia, it is estimated that the mortality rate exceeds 50% of the cases diagnosed with pediatric ALL [7].

Currently, the definitive diagnosis of ALL is carried out by cytogenetic and immunophenotype analysis in a bone marrow sample, in a stage where the disease has clearly manifested [8]. So far there is no early diagnostic test for ALL, which would be very useful in countries where access to health may be limited. Thus, we are interested in having a biomarker in peripheral blood against a bone marrow aspirate is a highly desirable objective and with great potential in the design of screening or screening tests.

The proteomics now provides a reliable alternative for the discovery of new biomarkers of diagnosis [9], the body fluids have great emerging potential in biomarker studies, especially those that can be collected by non-invasive or minimally invasive means (9–11). For example, the blood is considered a complex fluid tissue that encompasses cells and extracellular fluid where the variety of possible candidate biomarkers is significant (10,12).

The aim of this study was to examine the B-ALL plasma proteome, since it is the leukemia subtype with the highest incidence in Colombia, focused on the search for potential biomarkers to facilitate early diagnosis of ALL, improving the opportunity of treatment.

## Materials and methods

### Patients and samples

Plasma samples were collected from six pediatric patients with B-ALL before induction therapy. None of the subjects had received any prior treatment (Table 1). The diagnosis for each patient was based on morphological, immunophenotypic, and genetic tests. The plasma samples used as controls were obtained from six healthy children in the same age range. Blood samples were collected into Vacutainer K3 EDTA tubes. Plasma was separated by centrifugation and subsequently aliquoted into 0.5-mL tubes for cryopreservation at −80 °C. Plasma protein concentrations were determined using a BCA protein assay (Thermo Scientific Rockford, lL, USA). The study was conducted with the approval of the Research Ethics Committee at the National University of Colombia and the National Institute of Cancer of Colombia (Protocol Number INC GT00035). Informed consents were obtained from the patients before the samples were collected for analysis.

**Table 1:** Clinical characteristics of B-ALL patients.

Clinical features	Control	B-ALL
Age (years)	6 (3–9)	4.5 (2–9)
Sex (male/female)	6/0	4/2
White Blood Cell (WBC) count x10^9^/L	-	3.89 (0.47–112.8)
Hemoglobin (g/dL)	-	8.3 (5.02–10.9)
%Blast Bone marrow	-	61 (35–80)

Data are presented as median (minimum and maximum range) unless the sex.

### Depletion of high-abundance proteins from plasma

To reduce albumin and IgG, the most abundant plasma proteins, 30 μl of plasma was depleted with ProteoPrep (Immunoaffinity Albumin and IgG Depletion kit, Sigma-Aldrich, St. Louis, MO, USA). The depletion column was equilibrated using the equilibration buffer provided in the kit, and plasma samples were depleted according to the instructions by the manufacturer. The amount of plasma protein collected was determined using a BCA protein assay (Thermo Scientific Rockford, IL, USA). The immunodepleted proteins were subjected to total protein precipitation through the addition of cold TCA to obtain a concentration of 10%. The protein solutions were then mixed and stored for 1 hour at -10 °C and subsequently high-speed centrifuged at 8500 g and 4 °C for 30 min. The obtained pellet was washed three times with an ice-cold 90:10 acetone-water mixture. The samples were then centrifuged at 11,000 g for 5 minutes at 4 °C, and the protein pellet was air-dried and stored at -70 °C until analysis.

### In-solution tryptic digestion

The resulting protein pellet was solubilized in 100 μL of 6 M urea in 50 mM NH_4_HCO_3_. Dithiothreitol (DTT) was added to a concentration of 5 mM and the samples were incubated for 30 min at 37 °C. Next, 20 mM iodoacetamide (IAA) was added to an in-solution concentration of 15 mM, and the samples were incubated for 30 min at room temperature; this incubation was followed by the addition of 20 μL of 15 mM DTT over 10 min, and the samples were held at room temperature for 4 h. The samples were then diluted to a urea concentration of <1 M by the addition of 550 μL of 25 mM NH_4_HCO_3_. Trypsin (Promega, USA) was next added in a 1:25 enzyme-to-protein ratio and the samples were incubated overnight at 37 °C. The following day, the samples were desalted using C18 macro spin columns (Nest Group) and dried by vacuum centrifugation.

### Nano-LC-MS/MS analysis

LC separation was done on a nano Proxeon Easy-nLCTM II HPLC (Thermo Scientific, Waltham, MA) with a Proxeon nanospray source. The digested peptides were reconstituted in 2% acetonitrile/0.1% trifluoroacetic acid and roughly 3μg of each sample was loaded onto a 100-micron x 25 mm Magic C18 100Å 5U reverse phase trap where they were desalted online before being separated on a 75-micron x 150 mm Magic C18 200Å 3U reverse phase column. Each sample was injected three times. Peptides were eluted using a gradient of 0.1% formic acid (A) and 100% acetonitrile (B) with a flow rate of 300nL/min. A 90-minute gradient was run with 5% to 35% B over 70 minutes, 35% to 80% B over 8 minutes, 80% B for 1 minute, 80% to 5% B over 1 minute, and finally held at 5% B for 10 minutes. Each of the gradients was followed by a 1h column wash. Mass spectra were collected at the Davis Proteomics Core, University of California (http://proteomics.ucdavis.edu), on an Orbitrap Q Exactive Plus mass spectrometer (Thermo Fisher Scientific) in a data-dependent mode with one MS precursor scan followed by 15 MS/MS scans. A dynamic exclusion of 15 seconds was used. MS spectra were acquired with a resolution of 70,000 and a target of 1 × 10^6^ ions or a maximum injection time of 30ms. MS/MS spectra were acquired with a resolution of 17,500 and a target of 5 × 10^4^ ions or a maximum injection time of 50ms. Peptide fragmentation was performed using higher-energy collision dissociation (HCD) with normalized collision energy (NCE) value of 27. Unassigned charge states, as well as +1 and ions >+5, were excluded from MS/MS fragmentation.

### Protein identification and quantification

The raw data generated from the LC-MS/MS analysis were examined using database searches performed with Proteome Discoverer version 1.4 (Thermo Fisher Scientific), cross-referenced with results from UniProtKB/SwissProt (2016_05). The search parameters were defined as a precursor mass tolerance of 20 ppm and a fragment mass tolerance of 0.6 Da. The enzyme used was trypsin and one missed cleavage site was allowed. The carbamidomethylating of cysteines was defined as a fixed modification, while protein N-terminal acetylation and methionine oxidation were defined as variable modifications for database searching. The percolator program implemented in Proteome Discoverer was used to calculate the false discovery rate (FDR) of the identified peptides, and only peptides with FDR <0.01 were considered.

Progenesis QI for proteomics (v 3.0, Nonlinear Dynamics, New Castle, UK) was used for the performance of ion intensity-based label-free quantification. The retention times of eluting peptides from all of the samples in the experiment were aligned to a selected reference run, and only MS/MS peaks with a charge of 2+ to 5+ were considered for the total number of features (signal at one particular retention time and m/z). Only the five most intense spectra per feature were included. After the alignment and feature filtering, the raw abundances of all features were normalized against the total intensity to correct for the experimental variations, and a comparison of features between groups was performed by one-way analysis of variance (ANOVA, P <0.05 for statistical significance). Type-I errors were controlled by FDR with the q value set at 0.02. The associated unique peptide ion intensities for a specific protein were then summed to generate an abundance value. Search results from Proteome Discoverer were imported into Progenesis LC-MS to combine peptide quantification and identification. Only unique peptides for a corresponding protein were used for quantification. The dataset from the Progenesis analysis is shown in Supporting Information (S1 and S2 Tables).

Statistical analysis was performed using the MSStats and ROTS packages in R statistics [10–12]. Samples were annotated into two respective conditions (Control and B-ALL). Each sample was run three times in LC-MS/MS. For more details, see Supporting Information (S3 and S4 Tables).

### Bioinformatic analysis

To further understand the biological relevance of the differentially expressed proteins, we performed functional enrichment analysis using ClueGO (13). ClueGO, a widely used Cytoscape plugin, facilitates the visualization of functionally related genes displayed as a clustered network and chart. UniProt IDs of differential proteins were analyzed using the default parameters, which specify via a right-sided hypergeometric test an enrichment correction method using a Bonferroni step-down. The “Function” analysis mode, the gene cluster list for *Homo sapiens*, and a kappa score of 0.4 were used; the evidence codes were set to “All”, and the networking specificity was set to medium (GO levels 3 to 8).

### Determination of plasma concentrations by ELISA

Plasma aggrecan core protein (PGCA), alpha-2-HS-glycoprotein (FETUA), coagulation factor XIII A chain (F13A) and gelsolin (GELS) protein concentrations were determined by enzyme-linked immunosorbent assay (ELISA) analysis and compared 10 new samples of both control and B-ALL. Detailed procedures were performed according to manufacturer instructions to measure PGCA (Aviva Systems Biology Cat.OKEH00570), FETUA (MyBiosource Cat. MBS175929), F13A (Aviva Systems Biology Cat. OKEH02738) and GELS (Aviscera Bioscience, INC. Cat. SK00384-01) levels to generate measurement standards. Standard curves were created with protein concentration on the y-axis and average absorbance on the x-axis. The results were then calculated using the standard curves and multiplied by the dilution factor. Student’s t-test was used to analyze differences in protein levels between the control and cancer groups. A P-value <0.05 was considered statistically significant.

## Results

### Twenty-five differently expressed proteins were identified in B-ALL patients

**Fig 1:**
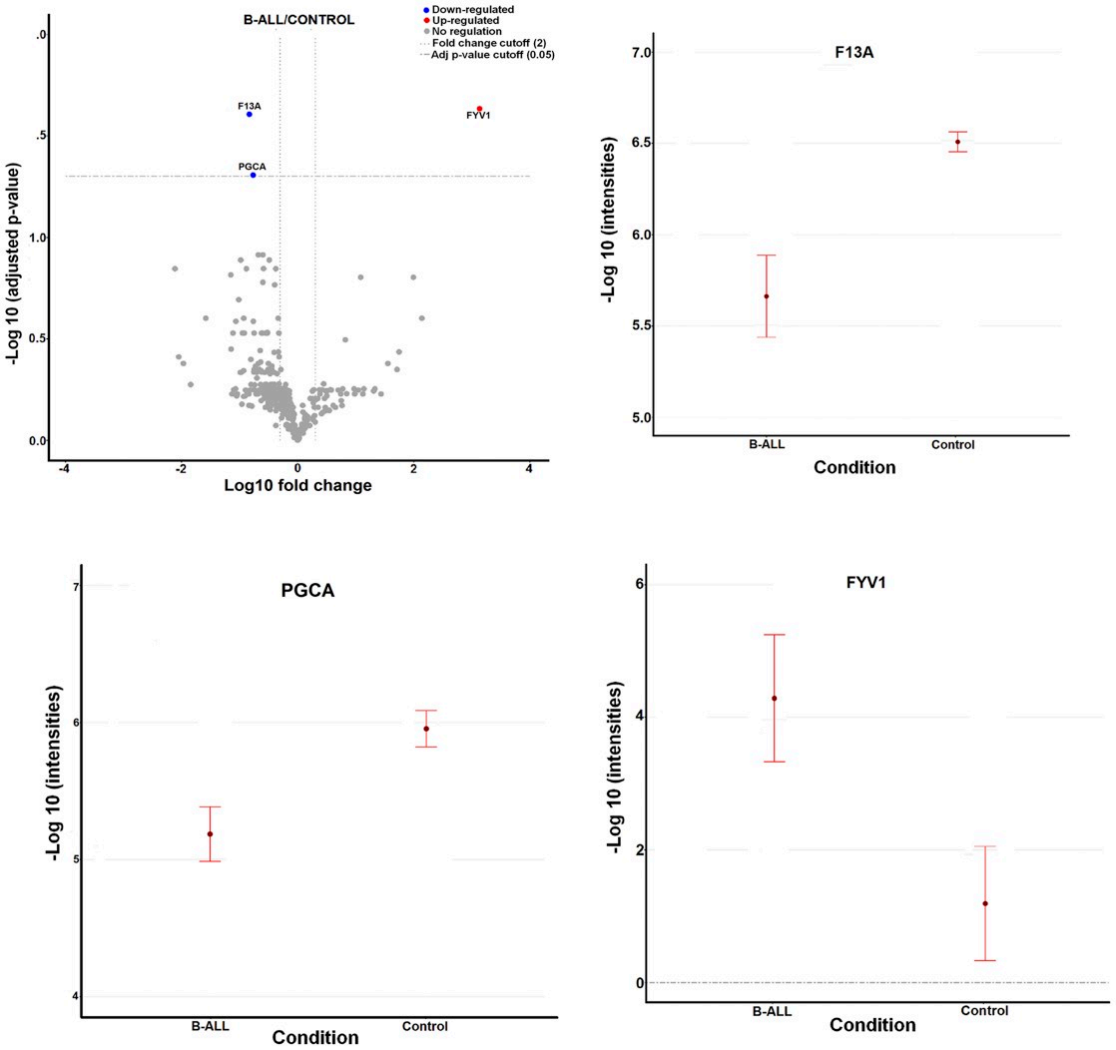
MSStats statistics. A. Volcano plot of the comparison B-ALL vs Control. The dashed line represents the false discovery rate (FDR) cutoff 5% and fold-change cutoff = 2.0. The up-regulated protein is shown in red dots and down-regulated in blue dots. Condition plots for FYV1 (B), PGCA (C) and F13A (D) proteins. Dots indicate the mean of log ratio and error bars have confidence intervals with 0.95 significant level for each condition.

In this study, we carried out a label-free proteome analysis using samples collected from six B-ALL patients and six healthy controls. A total of 472 proteins were quantified in the label-free analysis. MSStats was used to detect variations in protein abundance between the studied groups from the peptide measurements database. The analysis was designed as a comparative experiment using the “Group Comparison” function, setting the conditions to “Control” and “B-ALL”. A unique identifier was assigned to each biological replicate and each run. The intensity value was defined as the total area of each feature without transformation (data shown in supporting information S3 and S4 Tables). The significant changes in protein abundance were found using a linear mixed model, and the raw P-values were adjusted by the Benjamin and Hochberg method [12]. Significantly differentially expressed proteins in B-ALL patients compared to controls (those with an adjusted P-value <0.05, a fold change >2 and an FDR of 5%) are shown in a volcano plot (x = Log10FC, y = -Log10 adjusted P-value) (Fig 1A). The results showed that F13A and PGCA were down-regulated, and FYV1 was up-regulated (Table 2); the corresponding condition plot graphs are shown in Fig 1B, 1C and 1D.

**Table 2:** Differentially expressed proteins between B-ALL and control groups.

Accession number	Gene name	Protein name	MSStats Log_10_Fold change LLA-B vs. Control	MSStats *P* BH	ROTS Log_2_Fold change Control vs. LLA-B	ROTS *P* BONF
Q9Y2I7	*FYV1*	1-phosphatidylinositol 3-phosphate 5-kinase	3.134	0.0232		
P16112	*PGCA*	Aggrecan core protein	-0.7718	0.0487	2.359	0.001
P07333	*CSF1R*	Macrophage colony-stimulating factor 1 receptor			2.021	0.001
P00488	*F13A*	Coagulation factor XIII A chain	-0.8375	0.0244	1.961	< 0.001
Q9HCU4	*CELR2*	Cadherin EGF LAG seven-pass G-type receptor			1.864	< 0.001
P54108	*CRIS3*	Cysteine-rich secretory protein 3			1.817	< 0.001
P23142	*FBLN1*	Fibulin-1			1.802	< 0.001
P04278	*SHBG*	Sex hormone-binding globulin			1.780	< 0.001
O00533	*NCHL1*	Neural cell adhesion molecule L1-like protein			1.770	< 0.001
P02679	*FIBG*	Fibrinogen gamma chain			1.605	< 0.001
Q9UJV3	*TRIM1*	Probable E3 ubiquitin-protein ligase MID2			1.604	< 0.001
P55290	*CAD13*	Cadherin-13			1.574	0.0175
P02675	*FIBB*	Fibrinogen beta chain			1.576	< 0.001
P06396	*GELS*	Gelsolin			1.405	< 0.001
P06276	*CHLE*	Cholinesterase			1.378	< 0.001
P05160	*F13B*	Coagulation factor XIII B chain			1.359	< 0.001
P02671	*FIBA*	Fibrinogen alpha chain			1.333	< 0.001
P51884	*LUM*	Lumican			1.256	< 0.001
P11597	*CETP*	Cholesteryl ester transfer protein			1.247	0.0095
P01717	*LV403*	Ig lambda chain V-IV region Hil			1.244	< 0.001
P04196	*HRG*	Histidine-rich glycoprotein			1.239	< 0.001
Q7Z7M0	*MEGF8*	Multiple epidermal growth factor-like domains protein 8			1.150	0.009
P02765	*FETUA*	Alpha-2-HS-glycoprotein			1.140	< 0.001
Q6UXB8	*PI16*	Peptidase inhibitor 16			1.135	0.029
O00187	*MASP2*	Mannan-binding lectin serine protease 2			1.112	0.0085

FDR: False Discovery Rate, P BONF: P adjusted Bonferroni, P BH: P adjusted Benjamini-Hochberg. Fold Change represents a log value

**Fig 2:**
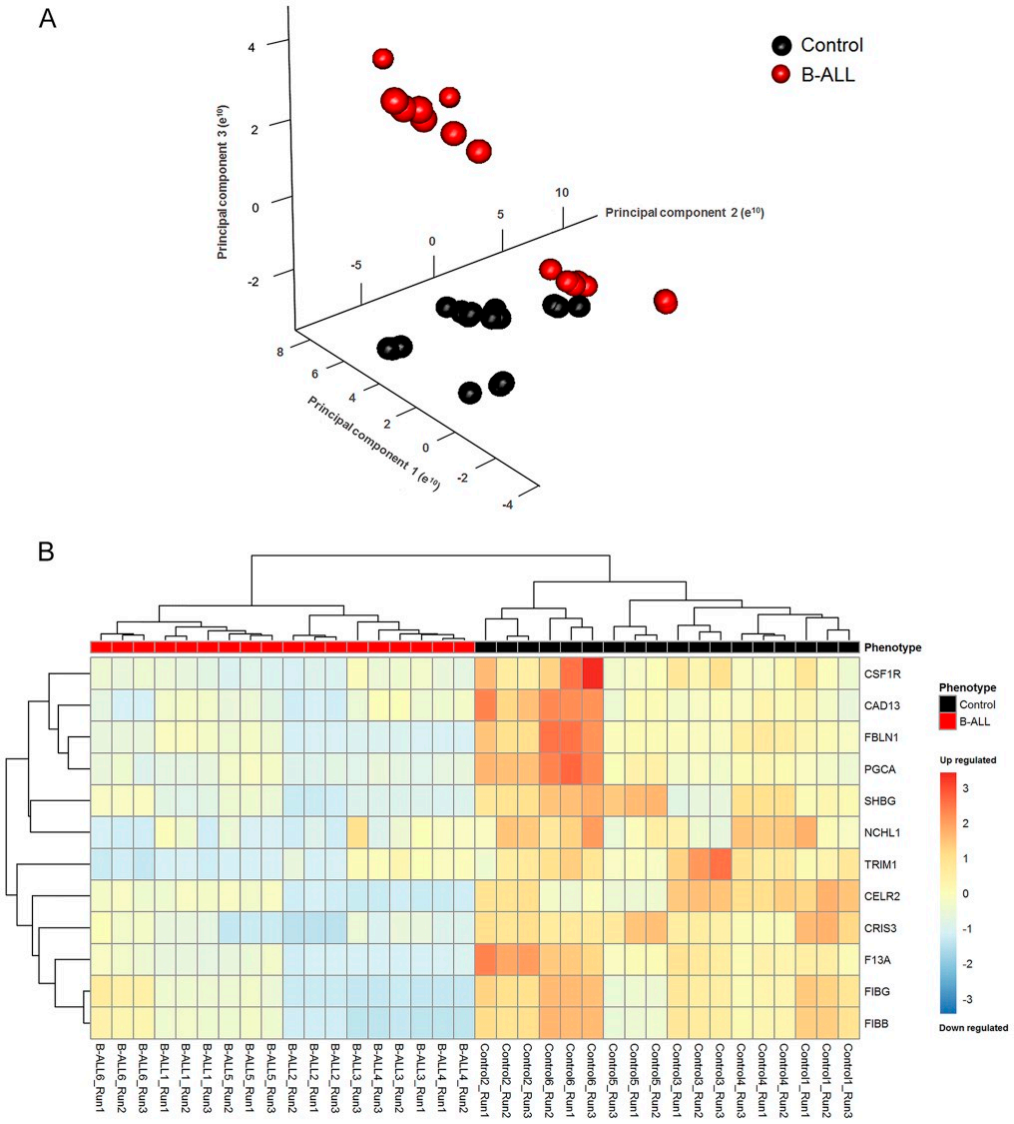
ROTS statistics. A. Unsupervised principal component score plot for controls and B-ALL patients. Red and black bubbles represent B-ALL and control plasma samples, respectively. The analysis resulted in a good separation between groups according to the principal component 1 (PC1) with 64,56% of explained variance, principal component 2 (PC2) 18,39% and principal component 3 (PC3) with 11,92%. B. Heat map of unsupervised clustering of the patients (columns) across the 12 proteins detected as differentially expressed by ROTS (rows), with a Bonferroni-adjusted P-value <0.05 and a fold change >3. Red and black bars represent B-ALL and control plasma samples, respectively.

ROTS classifies proteins according to evidence by means of examining the differential of expression in two-group comparisons [11,13]. For this analysis, the database “export protein measurements” was used and a 1000-repetition bootstrapping process was performed [11,14]. With the resulting enriched database, a multivariate analysis was performed using principal component analysis (PCA) to reveal larger variations (Fig 2A). A heatmap corresponding to differentially expressed proteins which had a fold change >2 and a Bonferroni-adjusted P-value <0.05 is shown in Supporting Information (data in S1 Fig). The 24 proteins identified provided a good separation between the groups, although the sample corresponding to control 5 did not match the overall control group. Fig 2B shows the heatmap corresponding to differentially expressed proteins with a Bonferroni-adjusted P-value <0.05 and a fold change >3. The number of proteins identified through these parameters was reduced from 24 to 12, but a complete separation between the groups was achieved.

### GO terms of differentially expressed proteins

**Fig 3:**
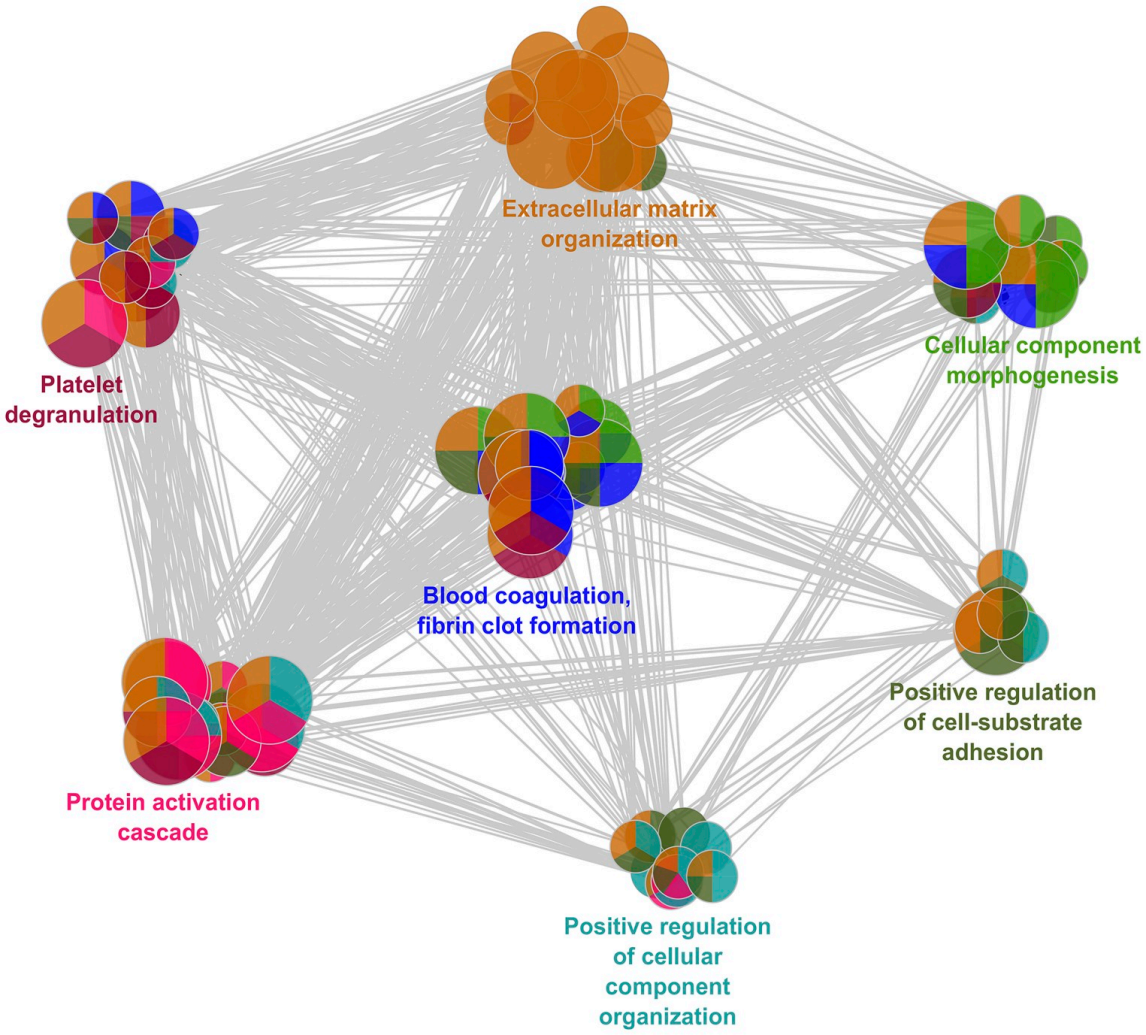
Grouping of networks based on functionally enriched GO terms. The functionally grouped network of enriched categories was generated for the differential proteins using ClueGO. GO terms are represented as nodes, the edges connecting the nodes are based on the kappa statistic that measures the overlap of shared genes between terms (less to 0.4). The node size indicates the number of proteins mapped to each term and the color represents the term enrichment significance. Only the most significant term in the group is labeled. Functionally related groups partially overlap. Visualization has been carried out using Cytoscape 3.4.0.

Overall, 69 GO terms were significantly enriched; these terms were categorized into seven GO groups as represented in Fig 3. The main GO categories were protein activation cascade, platelet degranulation, blood coagulation-fibrin clot formation, extracellular matrix organization, cellular component morphogenesis, and cell morphogenesis involved in the differentiation and positive regulation of cell-substrate adhesion.

### Plasma concentrations of PGCA, FETUA, F13A, and GELS proteins

**Fig 4:**
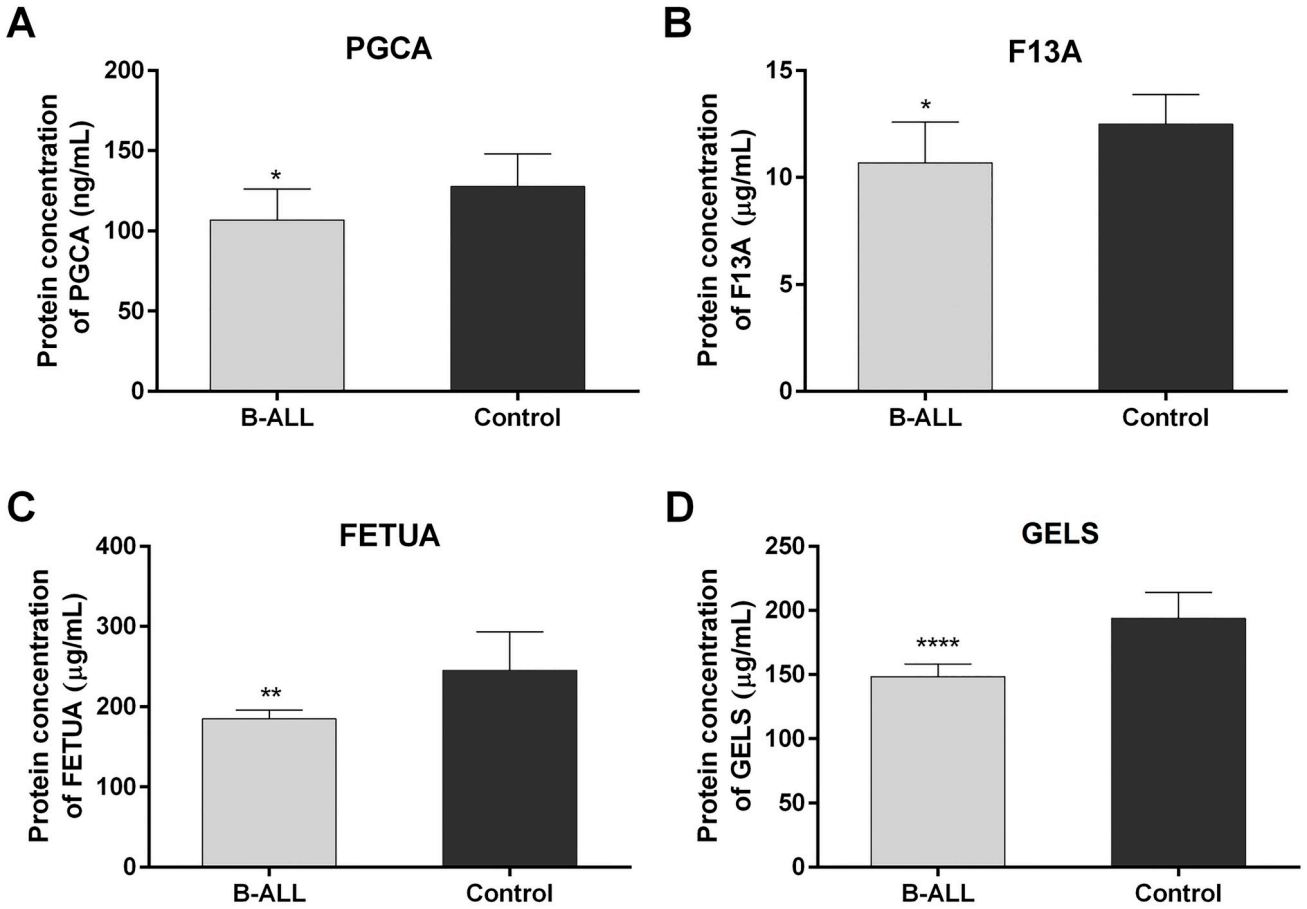
Plasma levels of PGCA, FETUA, F13A, and GELS. Plasma levels of PGCA (A), FETUA (B), F13A (C) and GELS (D) was detected by ELISA in B-ALL patients (n = 10) and control groups (n = 10). Statistical significance was determined by two-tailed non-paired Student’s t-test. Data as mean ± SEM. The analysis was performed using GraphPad Prism 7.0 **P <0.01 is significant, ****P <0.0001 is highly significant.

The plasma levels of PGCA, FETUA, F13A, and GELS were measured in plasma samples from individuals with B-ALL and healthy controls by ELISA (n = 10). The results showed that B-ALL patients had lower plasma concentrations of these proteins compared to those of the control group (Fig 4): (B-ALL patients vs controls) PGCA 106.7 ± 4.844 ng/mL vs 127.6 ± 4.985 ng/mL, FETUA 184.7 ± 3.111 μg/mL vs 245.2 ± 11.14 μg/mL, F13A 10.68 ± 0.4860 μg/mL vs 12.48 ± 0.3921 μg/mL and gelsolin 148.4 ± 3.005 μg/mL vs 193.7 ± 5.673 μg/mL (data shown in supporting information S5 Table). These results were consistent with the label-free quantitation measurements. Although there was a difference in the magnitude of the change between groups probably associated with the difference in sensitivity and the discriminating capacity of the immunoassay, the trends were in agreement in the two techniques.

## Discussion

Blood plasma is a biological fluid that is highly attractive for use in screening tests and biomarker discovery due to its ease of collection and its reflection of physiological changes produced by or in response to disease [15]. In this study, a proteomic approach was used to identify differentially expressed proteins in the blood plasma of patients diagnosed with B-ALL versus healthy controls. The blood plasma was depleted for two of the most abundant proteins, albumin, and IgG. Next, the plasma was analyzed by LC-MS/MS and quantified by label-free measurement. After statistical analysis, 25 proteins were identified as being differentially expressed. A decreased expression level was observed for 24 of these proteins, while the protein lipidic kinase 1-phosphatidylinositol-3-phosphate 5-kinase (PIKfyve or FYV1) was expressed at higher levels in patients with B-ALL than in healthy controls. This type-III PIP kinase participates in endosomal processes, autophagy regulation and exosome liberation [16]. PIKfyve controls phosphatidylinositol (PtdIns) (3,5) P2 levels to trigger the action of the SAC3 phosphatase to regenerate PtdIns3P or of the MTMR3 phosphatase to generate PtdIns5P. PtdIns5P is an intermediate that regulates the PI3K/Akt pathway and has been postulated as a second messenger in cellular migration, which suggests a role in oncogenesis [17]. Oppelt *et al* showed that FYV1 and MTMR3 are expressed in most cancerous cells and that decreased levels of these proteins produce an alteration of cell migration and are involved in the invasive behavior of cancer cells [18].

The hierarchical grouping produced in our analysis showed a group separation among the decreased differentially expressed proteins. The 12 proteins that presented a Bonferroni-adjusted P-value <0.05 and a fold >3 were interestingly grouped, given that they allowed a complete separation of the patients and the controls. We postulated that quantification of these proteins may be applied to achieve identification of B-ALL patients.

The functional annotation analysis showed that several of the identified proteins are involved in the blood coagulation process and have been reported as down-regulated in leukemia cases. For example, the coagulation factor F13A is a plasma transglutaminase that increases the coagulum resistance to fibrinolysis by forming covalent bonds between adjacent fibrine monomers and interweaving plasmin with fibrin inhibitors. Our proteomic approach showed that the F13A concentration was decreased in the blood plasma of patients with B-ALL. This observation has also been documented in whole plasma by ELISA assay in a study by Shi and Wang, in which a decrease of F13A in patients with acute myeloid and lymphoblastic leukemia and in solid tumors was reported [19]. Normal lymphoid cells do not synthesize or contain any F13 subunits, while lymphoid cells in bone marrow samples derived from patients with acute leukemia F13A do. This indicates F13A as a possible intracytoplasmic marker that can be used in the identification of aberrant phenotypes, which could provide a valuable diagnostic tool for the identification of acute leukemia from myeloid and B-lymphoid lineages [20].

Fibrinogen, another important member of the coagulation cascade, undergoes altered expression in different types of cancer, and it has been reported that the expression of FGA is increased in serum from ALL patients [21]. However, we observed a decreased level of all three-polypeptide chains (α, β and γ) probably due to a common thrombocytopenia stage in the leukemic patients.

A second identified group of proteins is involved in adhesion, extracellular matrix organization, and cell morphogenesis processes. Of these, fibulin 1, gelsolin, histidine-rich glycoprotein, cadherin EGF LAG seven-pass G-type receptor 2 and cadherin-13 are highlighted and discussed below.

Fibulin 1, an extracellular matrix protein, and gelsolin, an actin-binding protein and key regulator in the assembling/disassembling of actin filaments, were found to be down-regulated in B-ALL samples. Down-regulation of these proteins has also been shown in other types of cancers, suggesting that they may function as tumor suppressors [22,23]. In leukemia U937 cells, gelsolin overexpression induced a retarded growth, an improved monocytic morphology, increased NADPH activity and enhanced superficial expression of the CD11b β-integrin receptor in comparison with U937 parental cells [24]. Our study showed concordant results with these previous findings. Gelsolin was decreased in plasma from B-ALL patients, a result that was confirmed by the ELISA measurement of the protein in whole plasma.

Interestingly, cadherin EGF LAG seven-pass G-type receptor (CELSR2) and cadherin-13 have not previously been reported in blood plasma, but they appear to be related to the malignancy processes of hematopoietic stem cells (HSC). These cadherins belong to the subfamily of Flamingo (Fmi) or CELSR cadherins and are involved in adhesion and cell-cell recognition processes [25]. These two cadherins intervene in the non-canonical Wnt pathway through binding with Frizzled (Fz) to regulate planar cell polarity and increase free intracellular calcium. Non-canonical Wnt signaling is necessary to maintain HSC quiescence by suppressing the Ca^2+^-nuclear factor signaling of activated T-cell (NFAT)-gamma-interferon (IFNγ) and the antagonist signaling of canonical Wnt. Fz8 and Fmi binding results in a non-canonical receptor of Wnt signaling, which response to non-canonical Wnt signaling as Wnt5a. Under stress conditions, canonical Wnt signaling activates HSCs to induce auto-renovation and differentiation. Non-canonical signaling by Wnt5a is increased in the new HSC population for a short term through the maintenance of HSCs in the quiescent phase (G0) [26,27]. The decreased expression of these two cadherins in B-ALL patients suggests an alteration of the Wnt signaling pathway to produce a change in the quiescence state of HSCs. Truncated cadherin-13 (T-cadherin) is an atypical member of the cadherin superfamily. It lacks transmembrane and cytoplasmic domains and is bound to the plasma membrane through a glycosylphosphatidylinositol anchor. T-cadherin has been suggested as a tumor suppressor, since in several types of cancer, including B-cell lymphoma and chronic myeloid leukemia (CML), it has been found to be down-regulated by allelic deletion and/or by promoter hypermethylation [28]. It is interesting to find these two related-to-non-canonical-Wnt-signaling cadherins decreased in B-ALL patients, we could speculate an alteration of this signaling pathway and thus a change of quiescence state of HSC.

Another highlighted protein is Aggrecan core protein (PGCA), a proteoglycan that is the main component of the extracellular matrix of cartilaginous tissue [29]. The extracellular matrix has been a focus of study in the examination of tumor invasion and metastasis, since these processes imply a complex series of events, including extra-cellular matrix proteolysis. PGCA is thought to be a tumor suppressor. In studies of larynx cancer, PGCA has presented a reduced expression and has been correlated with a worse prognosis and an increased cancer metastasis rate. PGCA homozygotic deletion is also related to the development of classic Hodgkin’s lymphoma induced by the *Epstein-Barr* virus [30]. PGCA plasma expression levels measured by ELISA confirmed the decreased expression of the protein in plasma from patients with B-ALL.

Lumican is a member of the small leucine-rich proteoglycan family, and it has been identified to undergo overexpression or negative regulation in different types of cancer. For instance, a decrease of 2–3 times the normal expression has been reported for lumican in breast cancer tissue. Additionally, lumican expression was further decreased in advanced disease stages than in early disease stages. Plasma proteomic analysis has demonstrated that lumican levels were significantly higher in patients with lung cancer in comparison with normal subjects [31]. Lumican is also implied in the negative regulation of the proteolytic activity associated with endothelial cell membranes, in particular, that of matrix metalloproteinases MMP-14 and MMP-9, as well as that of MMP-1, MMP-2, and MMP-13 [32]. Lumican blocks the migration and invasion of tumor cells. In general, lumican seems to be a potent agent for the inhibition of tumor progression, which is interesting given that MMP-2 and MMP-9 expression has been reported as positively altered in ALL cases [33,34].

CSF1R, also known as the receptor for the macrophage colony-stimulating factor (M-CSFR), is a member of the class-III tyrosine kinase receptor family. Along with its ligand CSF1L, it has a critical role in cell survival regulation and in the myeloid cell differentiation involved in macrophage lineage as well as bone formation. In adult hematopoiesis, CSF1R is selective and highly expressed in myeloid and dendritic cells but is not detectable in lymphocytes, including progenitor and mature B-cells. It has been suggested that there exists a connection between B-lineage development and myeloid lineages during fetal development, which may explain the chromosomic translocations of the mixed lineage leukemia (MLL) gene observed in breastfed infants diagnosed with B-ALL [35]. The CSF1R expression results are particularly interesting due to its role in a translocation observed in high-risk patients with a Philadelphia-like chromosome (Ph-like)^+^. Translocation t(1; 5) (q21; q33) has been reported between *CSF1R* and *MEF2D* in one ALL case [36], and the fusion of *CSF1R* with *SSBP2*, *TBL1XR1* genes has also been reported [37].

Other differentially expressed protein related to diverse cell metabolic processes was the fetuin-A, also down-regulated in B-ALL patients. Fetuin-A is a phosphorylated glycoprotein with multipotent properties which plays an anti-inflammatory role by counteracting pro-inflammatory cytokine production. Like haptoglobin, fetuin-A is an acute-phase protein, for which the serum concentration diminishes in comparison with normal levels as a response to inflammation. It is important to highlight that acute-phase proteins increase blood flow to the tumor microenvironment, and they may be relevant in tumorigenesis processes. Acute-phase proteins can also reflect a systemic inflammatory response. Fetuin-A is synthesized and secreted predominantly by the liver in adults. It is also involved in calcium homeostasis and in the decrease of insulin signaling through inhibition of the phosphorylation of insulin tyrosine-kinase receptors. Fetuin-A has been proposed as a potential tumor marker in specific malign neoplasia, including in pancreatic and breast cancer, and a decrease in Fetuin-A levels in serum from patients with chronic lymphoblastic leukemia (CLL) compared to those of healthy controls has been reported [38]. In proteomic studies, fetuin-A has been shown to be decreased in several cancer types. Tian showed a fetuin-A decrease in plasma from patients with hypopharyngeal squamous cell carcinoma (HSCC) [39]. Dowling reported decreased fetuin-A levels in serum from breast cancer patients compared to those of healthy controls [40]. Kwak also showed a fetuin-A decrease in serum from acute myeloid leukemia (AML) patients in comparison with healthy controls [41]. Our results also indicated lower fetuin-A expression levels in plasma from patients with B-ALL in comparison with those of healthy controls via both the proteomic and ELISA approaches, which further indicates the potential of fetuin-A as a possible tumor marker.

## Conclusion

This study makes public a first approximation of the alterations in the plasma proteome of Colombian B-ALL patients. We report some potential biomarkers that could be used to differentiate unhealthy patients from healthy individuals; these biomarkers would aid treatment providers in one of the most complex and difficult clinical decisions in relation to certain age groups. This exploratory work paves the way for new studies using the identified differentially expressed proteins for further clinical development. To validate the specificity and the potential applicability of our proposed markers, it is necessary to perform a further study involving a population of greater size and diversity.

## Supporting information

S1 Table Quantification results using Progenesis QI: Normalized intensities by proteins. (XLSX)

S2 Table Quantification results using Progenesis QI: Normalized intensities by peptides. (XLSX)

S3 Table Data results of MSStats statistical package. (XLSX)

S4 Table Data results of ROTS statistical package. (XLSX)

S5 Table Data results of ELISA assay. (XLSX)

S1 Fig Heat map of unsupervised clustering of the patients across the 12 proteins detected as differentially expressed by ROTS. Bonferroni-adjusted P-value <0.05 and a fold change >2. Red and black bars represent B-ALL and control plasma samples, respectively.
(TIF)
