# Supplementary figures and images for: A comparative proteomic study of plasma in Colombian childhood acute lymphoblastic leukemia
Source: PLoS One. 2019 Aug 22;14(8):e0221509. doi: 10.1371/journal.pone.0221509 (PMC6705836; doi:10.1371/journal.pone.0221509)

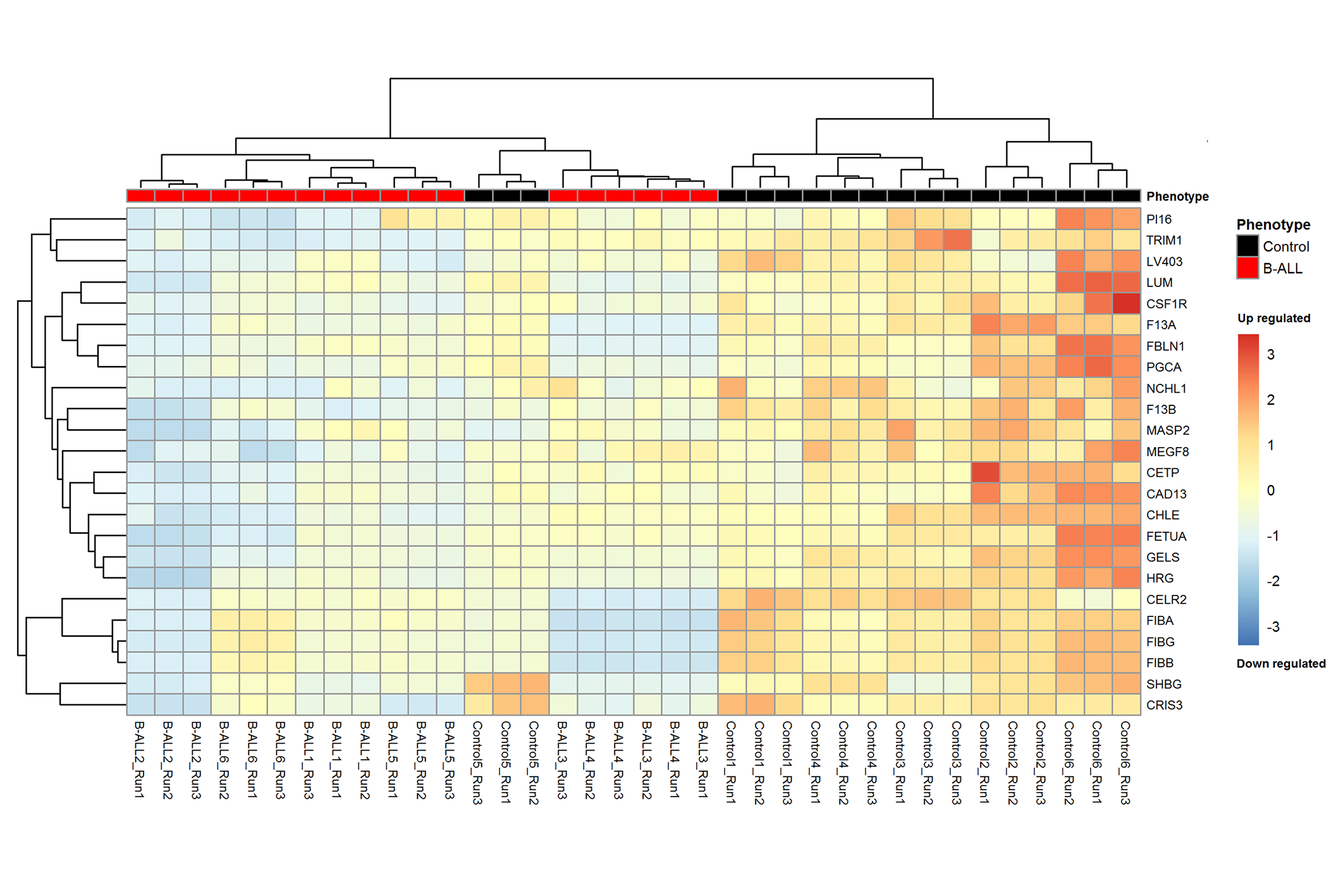

Supplement: S1 Fig — Bonferroni-adjusted P-value <0.05 and a fold change >2. Red and black bars represent B-ALL and control plasma samples, respectively. (TIF) [file pone.0221509.s006.tif]
